# Cross-reactive humoral and CD4^+^ T cell responses to Mu and Gamma SARS-CoV-2 variants in a Colombian population

**DOI:** 10.3389/fimmu.2023.1241038

**Published:** 2023-07-27

**Authors:** Fabiola Martel, Juliana Cuervo-Rojas, Juana Ángel, Beatriz Ariza, John Mario González, Carolina Ramírez-Santana, Yeny Acosta-Ampudia, Luisa Murcia-Soriano, Norma Montoya, Claudia Cecilia Cardozo-Romero, Sandra Liliana Valderrama-Beltrán, Magda Cepeda, Julio César Castellanos, Carlos Gómez-Restrepo, Federico Perdomo-Celis, Andreu Gazquez, Alexandria Dickson, James D. Brien, José Mateus, Alba Grifoni, Alessandro Sette, Daniela Weiskopf, Manuel A. Franco

**Affiliations:** ^1^ Institute of Human Genetics, School of Medicine, Pontificia Universidad Javeriana, Bogotá, Colombia; ^2^ Department of Clinical Epidemiology and Biostatistics, School of Medicine, Pontificia Universidad Javeriana, Bogotá, Colombia; ^3^ Clinical Laboratory Science Research Group, Clinical Laboratory, Hospital Universitario San Ignacio, Bogotá, Colombia; ^4^ Group of Basic Medical Sciences, School of Medicine, Universidad de Los Andes, Bogotá, Colombia; ^5^ Center for Autoimmune Diseases Research (CREA), School of Medicine and Health Sciences, Universidad del Rosario,, Bogotá, Colombia; ^6^ Hospital Universitario Mayor-Méderi, Universidad El Rosario, Bogotá, Colombia; ^7^ Head Clinical Laboratory Unit, Clínica del Occidente, Bogotá, Colombia; ^8^ Division of Infectious Diseases, Department of Internal Medicine. School of Medicine, Pontificia Universidad Javeriana, Hospital Universitario San Ignacio Infectious Diseases Research Group, Bogotá, Colombia; ^9^ General Direction, Hospital Universitario San Ignacio, Bogotá, Colombia; ^10^ Department of Molecular Microbiology and Immunology, Saint Louis University School of Medicine, St. Louis, MO, United States; ^11^ Center for Infectious Disease and Vaccine Research, La Jolla Institute for Immunology, La Jolla, CA, United States; ^12^ Department of Medicine, Division of Infectious Diseases and Global Public Health, University of California, San Diego, La Jolla, CA, United States

**Keywords:** SARS-CoV-2, variants, natural infection, vaccination, antibody, CD4+ T cell, hybrid immunity, breakthrough infections

## Abstract

The SARS CoV-2 antibody and CD4^+^ T cell responses induced by natural infection and/or vaccination decline over time and cross-recognize other viral variants at different levels. However, there are few studies evaluating the levels and durability of the SARS CoV-2-specific antibody and CD4^+^ T cell response against the Mu, Gamma, and Delta variants. Here, we examined, in two ambispective cohorts of naturally-infected and/or vaccinated individuals, the titers of anti-RBD antibodies and the frequency of SARS-CoV-2-specific CD4^+^ T cells up to 6 months after the last antigen exposure. In naturally-infected individuals, the SARS-CoV-2 antibody response declined 6 months post-symptoms onset. However, the kinetic observed depended on the severity of the disease, since individuals who developed severe COVID-19 maintained the binding antibody titers. Also, there was detectable binding antibody cross-recognition for the Gamma, Mu, and Delta variants, but antibodies poorly neutralized Mu. COVID-19 vaccines induced an increase in antibody titers 15-30 days after receiving the second dose, but these levels decreased at 6 months. However, as expected, a third dose of the vaccine caused a rise in antibody titers. The dynamics of the antibody response upon vaccination depended on the previous SARS-CoV-2 exposure. Lower levels of vaccine-induced antibodies were associated with the development of breakthrough infections. Vaccination resulted in central memory spike-specific CD4^+^ T cell responses that cross-recognized peptides from the Gamma and Mu variants, and their duration also depended on previous SARS-CoV-2 exposure. In addition, we found cross-reactive CD4^+^ T cell responses in unexposed and unvaccinated individuals. These results have important implications for vaccine design for new SARS-CoV-2 variants of interest and concern.

## 1 Introduction

The COVID-19 pandemic, caused by the Severe Acute Respiratory Syndrome Coronavirus 2 (SARS-CoV-2), has been present for more than three years, resulting in widespread morbidity and mortality throughout the world (1). One of the main measures to mitigate the impact of COVID-19 has been the generation of vaccines targeting conserved viral epitopes. Most of the current vaccine platforms that target the Spike (S) protein of SARS-CoV-2, are based on the ancestral Wuhan variant, and aim to induce virus-specific antibodies and T cells (1–9). However, new viral variants may be differentially recognized by the immune system, leading to decreased protection after natural infection and/or vaccination (10–12). While current COVID-19 vaccines are highly immunogenic and effective at preventing severe disease, several questions remain unanswered (1): a) How do COVID-19 vaccines induce protection and how can we asses this protection (i.e. what are the correlates of protection); b) How effective are COVID-19 vaccines against viral variants of concern (VOCs) or variants of interest (VOIs), and at preventing breakthrough infections (BTI); c) Are vaccine-mediated protection and its durability determined by the pre-vaccination history of natural infection and/or the population´s geographic region?

Although several studies have evaluated the presence of neutralizing antibodies to viral variants (11–15), there are no studies evaluating both the humoral and cellular responses against the Mu variant, which circulated widely in Colombia in 2021 and has been shown to evade the humoral response (12). In this regard, previous studies in US, British and Chinese populations have observed that the T cell response is conserved for several variants after natural infection and vaccination (3, 10, 16), but this is not clear for the Mu variant. Moreover, it is incompletely known whether immunity generated by vaccination and/or natural infection with the ancestral variant persists over time and whether it is effective in preventing BTI with new viral variants (4, 11, 16).

An important aspect to consider regarding vaccine-induced immunity against SARS-CoV-2 is preexisting immunity (both previous natural exposure to SARS-CoV-2, and cross-reactive responses to human “common cold” coronaviruses [HCoVs]). It has been observed that multiple SARS-CoV-2 exposures enhance the magnitude of IgG responses but cause a low impact on the frequency of S-specific T cells (1). However, these T cells resulting from hybrid immunity may exhibit a wider polyfunctional profile than those from vaccination alone (17). Thus, the impact of previous antigen exposure on the levels and durability of humoral and cellular responses and the interplay of viral variants remain to be fully evaluated.

In this study, we evaluated the levels and durability of the receptor binding domain (RBD)-specific antibody and the CD4^+^ T cell responses induced by natural infection and/or vaccination against SARS-CoV-2 Wuhan, Gamma, Mu, and Delta variants, as well as the impact of disease severity and the history of SARS-CoV-2 exposure on such responses. Overall, our results indicate that both natural infection and vaccination induce cross-reactive immune responses for the Gamma, Mu, and Delta variants, and their levels, durability, and or quality, are influenced by sequential antigen exposure.

## 2 Methods

### 2.1 Cohorts of naturally infected and vaccinated individuals

To assess the antibody response against SARS-CoV-2 Wuhan, Gamma, Mu, and Delta variants, we assembled two ambispective cohorts in Bogotá, Colombia, with adult donors from the Universidad de Los Andes, Hospital Universitario San Ignacio (HUSI) recruited by Pontificia Universidad Javeriana, and from Hospital Universitario Mayor-Méderi and Clínica del Occidente, these last two being recruited by Universidad del Rosario. Cohort 1 is composed of naturally infected individuals who underwent follow-up and antibody measurements at approximately 1- and 6 – 8 months post-symptoms onset (PSO) to determine the seropermanence of antibodies induced by natural infection. Cohort 2 is composed of vaccinated individuals, in whom measurements of antibodies were performed during the pre-vaccination, and at the early (15 – 30 days after the second dose), and late post-vaccination periods (6 – 8 months and 12 months after the second dose), to determine the seropermanence of antibodies induced by vaccination. A fraction of the individuals also received a third vaccine boost and a sample was also obtained 1-3 months after this dose. The CD4^+^ T cell response was evaluated in a subgroup of individuals belonging to cohort 2.

### 2.2 Samples

For serological assays, whole blood was collected in clot activator and gel separation tubes, and serum was collected and stored at -80°C. To evaluate the CD4^+^ T cell response, whole blood was collected in EDTA tubes, and the cellular fraction was separated from plasma by centrifugation at 10,000 rpm for 10 minutes. Next, peripheral blood mononuclear cells (PBMCs) were isolated by density gradient centrifugation using Ficoll-Paque™ (Cytiva). Isolated PBMCs were cryopreserved in cell recovery media containing 90% fetal bovine serum (FBS) and 10% DMSO (ATCC) and stored in liquid nitrogen until cellular assays were performed.

### 2.3 Chemiluminescent assay

To evaluate if an individual was naturally infected by SARS-CoV-2, an IgG test that recognizes the viral nucleoprotein was performed (09203095190 – ROCHE), using a Roche Cobas e analyzer, following the manufacturer’s protocol. The positive and negative controls were performed at the beginning of each lot of antibody testing for quality control. Samples with a cutoff index (COI) ≥ 1 were considered positive. This assay was performed in a commercial clinical laboratory.

### 2.4 Hemagglutination test

To assess the presence of RBD-specific antibodies induced by natural infection or vaccination against SARS-CoV-2 Wuhan, Gamma, Mu, and Delta variants, a hemagglutination Test (HAT) that measures total antibodies (IgG, IgM, IgA) was employed (18, 19). O-negative red blood cells from a healthy volunteer were isolated from whole blood by density gradient centrifugation according to the manufacturer’s instructions. Red blood cells were preserved in Alsever’s solution (Sigma Aldrich) at 2°C for up to 30 days. After standardization, the HAT was performed using red blood cells from the same volunteer. For the HAT, codon-optimized IH4-RBD sequences of VOC and VOIs containing amino acid changes in the RBDs Gamma (K417T, E484K, N501Y), Mu (R346K E484K N501Y), and Delta (L452R, T478K) were expressed in Expi293F cells and purified by their c-terminal 6xHis tag using Ni-NTA chromatography (18, 19). The point HAT was performed in a V-bottomed 96-well plate. Serum samples were double diluted in duplicate from 1:40 in 50 µl of phosphate-buffered saline (PBS), up to 1:1,280. Samples with titers >1:1,280 were re-tittered until negative (1:10,240). Equal volumes of human O-negative red blood cells and 1 µg/mL of IH4-RBD of Wuhan, Gamma, Mu, and Delta variants were premixed, adding 100 µL per well, as described in our previous publication (20). Plates were incubated for 1 hour at room temperature to allow red blood cells to settle; then, the plate was tilted for 30 seconds, read, and photographed. Positive agglutination HAT titer (here reported as the reciprocal titer) was defined as the last dilution in which the teardrop did not form. Partial teardrops were considered negative (18). A representative example of HAT is shown in **Supplementary Figure 1**. To validate the assay, we tested reference serum samples with different levels of antibodies against SARS-CoV-2, the serum 20/130 provided by WHO, as well as the monoclonal antibodies CR3022 and EY-6A (kindly provided by Dr. Alain Townsend), with expected results (20).

### 2.5 Focus reduction neutralization assay

Serum samples were serially diluted in DMEM containing 5% FBS and combined with ~100 focus forming units (FFU) of SARS-CoV-2 variants Mu (BEI#NR-56225) and Gamma (BEI#NR-54982) and allowed to complex at 37°C and 5% CO2 for 1 hour in a 96-well round bottom plate. The antibody-virus complex was then added to each well of a 96-well flat bottom plate containing a monolayer of Vero-hACE2-TMPRSS2 cells. Following 1 hour of incubation at 37°C and 5% CO2, the cells were overlaid with 2% methylcellulose and returned to the incubator. After 24 hours of infection, the cells were fixed with 5% electron microscopy grade paraformaldehyde in PBS for 15 minutes at room temperature. The cells adherent to the plate were then rinsed with PBS and permeabilized with 0.05% Triton-X in PBS (FFA Wash Buffer). Foci of infected Vero-hACE2-TMPRSS2 cells were stained with anti-SARS-CoV-2 human mAb 2165 (Leinco) overnight at 4°C and washed 3 times with FFA Wash Buffer. Cells were then stained with horseradish peroxidase-conjugated goat anti-human IgG for 2 hours at room temperature. Cells were washed again with 0.05% FFA Wash Buffer prior to the addition of TrueBlue KPL peroxidase substrate, which allows the visualization of infected foci as blue spots. The foci were visualized and counted using an ImmunoSpot CTL Elispot plate reader. Percent infection was calculated by the number of foci per well/number of foci in negative control wells*100.

### 2.6 SARS-CoV-2 peptide mega pools

Spike mega pool (CD4S) sets consisting of 253 overlapping peptides of 15-mer with 10 amino acid overlap were synthesized to span the entire S protein of the Wuhan, Gamma, and Mu variants sequences, as previously reported (4). In addition, for the rest of the Wuhan variant proteome (CD4R), a mega pool of 221 dominant HLA class II predicted epitopes from the Immune Epitope Data Base (IEDB) was used to assess the complete response against the SARS-CoV-2 proteome, excluding the S protein.

### 2.7 Flow cytometry-based T cell assays

The Activation-Induced Markers (AIM) assay has been previously described in detail (4). The PBMCs that were preserved in liquid nitrogen were thawed by diluting the cells in 10 mL of complete RPMI 1640 (Invitrogen) with 5% human AB serum (Gemini Bioproducts) in the presence of benzonase-nuclease (Merck Millipore). The PBMCs were cultured in the presence of SARS-CoV-2 Wuhan, Gamma, and Mu variants peptide mega pools (at 1 µg/mL) in 96 well U bottom plates, at 1x10^6^ PBMCs/200 µL/well. DMSO was used as a negative control, while Staphylococcal Enterotoxin B (SEB, at 4 µg/mL) was used as a positive control. Cells were stimulated for 24 hours at 37°C, and 5% CO_2_. Upon incubation, cells were washed with PBS, and stained for 20 minutes at 4°C with the viability marker Live/Dead Aqua (Invitrogen, at 1/40 dilution). Next, the cells were washed with PBS, and incubated for 30 minutes at 4°C with fluorochrome-labeled antibodies against human CD19, CD14, CD3, CD4, CD8, CD134 (OX40), CD137, CD45RA, and CCR7 (**Supplementary Table 1**). Next, the cells were washed with FACS buffer (PBS and 3% FBS) and fixed with paraformaldehyde 1%. Cells were acquired on an Aurora spectral flow cytometer (Cytek) and analyzed in SpectroFlo software (v.3.0). Gates for AIM^+^ cells (OX40^+^ CD137^+^) were set according to the negative and positive controls for each donor. A representative example of the gating strategy is shown in **Supplementary Figure 2**. For the resulting AIM data, the background of AIM^+^ cells in the DMSO condition of each sample was removed, with a minimal DMSO level set to 0.005%. A positive response was defined as a stimulation index (SI) >2, and above 2 times the standard deviation of DMSO values (6). In SEB-stimulated cells from all the samples included the geometric mean (95% CI) was 3.89% (3.38-4.47%).

### 2.8 Statistical analysis

Data were analyzed and plotted in GraphPad Prism software, version 9.3.1. In all the cases, the geometric mean and 95% confidence intervals (CI) are shown. To determine statistical differences between the groups studied, non-parametric tests were performed. The Mann-Whitney and Wilcoxon tests were used for the comparison of two unpaired and paired groups, respectively. The Kruskal-Wallis and Friedman tests were used for the comparison of three or more unpaired or paired groups, respectively. The Dunn test was applied to correct for multiple comparisons. In all the cases, a p-value <0.05 was considered as significant.

## 3 Results

### 3.1 Cohorts of naturally infected and vaccinated donors

In this study, 549 participants were enrolled and classified into naturally infected (**Table 1**) and vaccinated cohorts (**Table 2**), according to medical records and study surveys. Naturally infected donors (n=113) included adults with a history of mild (Javeriana and Andes participants from cohort 1) and severe hospitalized (Rosario participants from cohort 1) SARS-CoV-2 infection confirmed by PCR and/or serologic tests. Samples from these individuals were collected at 1- and 6-month PSO (**Table 1**). The vaccinated cohort has donors with and without a history of natural SARS-CoV-2 infection. The part of the cohort recruited by Javeriana (HUSI from cohort 2) had individuals vaccinated with two doses of the BNT162b2 vaccine, in whom samples were collected at pre-vaccination, at 15-30 days, and 6-8 months after the second vaccine dose (**Table 2**). The part of cohort 2 recruited by El Rosario- consisted of individuals who, in addition to two doses of the BNT162b2 vaccine, also received a third dose of mRNA-1273, ChAdOx1 nCoV-19, or Coronavac vaccines (approximately 10 months after the second vaccine dose). Samples from these donors were collected 15-30 days after the second vaccine dose, and 1-3 months after the third dose (**Table 2**). A fraction of these individuals also had pre-vaccination samples (**Table 2**). Nucleoprotein-specific IgG antibodies were assessed to determine asymptomatic and BTI in the vaccination cohort, as previously reported (17). The characteristics of the subgroup of individuals belonging to cohort 2, to whom the CD4^+^ T cell response was evaluated, are summarized in **Supplementary Table 2**.

**Table 1:** Demographic characteristics of the natural infection cohort.

Cohort	Javeriana		Andes			Rosario	
Time point	1 month PSO	6 months PSO	1 month PSO	6 months PSO	6 month-follow-ups negative at baseline *	1 month PSO	6 months PSO
**Number of donors**	12	12	26	21	29	13	
**Age**	36 (26–50)	32 (26 – 47)	36.6 (21 – 59)	38 (21 – 59)	37 (23 – 57)	52.6 (40 – 67)	
**% Sex (M/F)**	(58/42)	(67/33)	(58/42)	(57/43)	(69/31)	(62/38)	
**Sample collection date**	June – July 2020	December 2020 – January 2021	November-December 2020	May – June 2021	May 2021	August – October 2020	March – April 2021
**Symptoms**	Mild		Mild			Severe	

* Individuals who were seronegative at baseline and seroconverted during recruitment period. PSO, Post-symptoms onset.

**Table 2:** Demographic characteristics of the vaccination cohort.

Cohort	Javeriana n= 224			Rosario				
				Sub-cohort 1 (CDO) n= 47		Sub-cohort 2 (Méderi) n=75		
Time point	Pre-vaccine	15-30 days after second dose¹	6-8 months after second dose	15-30 days after second dose	1-3 months after third dose²	Pre-vaccine	15-30 days after second dose	1-3 months after third dose³
**Number of** **donors**	68	213	224	47	44	75	73	38
**Age**	37.3 (19 – 52)	40.1 (19 – 64)	39.9 (19-64)	43 (22 – 60)	43 (22 – 60)	36.8 (22 – 57)	37 (22 – 57)	39 (25 – 56)
**% Sex (M/F)**	(12/88)	(17/83)	(17/83)	(23/77)	(25/75)	(35/65)	(36/64)	(24/75)
**Sample collection date**	March 2021	April – June 2021	October – November 2021	May 2021	February 2022	November 2020	April 2021	April 2022
**# of positive Nucleoprotein antibody responders**	28	72	84	23	25	ND	24	ND

¹All individuals received the BNT162b2 vaccine. ² Vaccines received: 33 mRNA-1273, 6 ChAdOx1 nCoV-19, and 5 BNT162b2. ³ Vaccines received: 36 mRNA-1273, 1 BNT162b2, and 1 Coronavac. CDO, Clínica de Occidente; ND, Not determined.

### 3.2 The duration of RBD-specific antibodies is associated with COVID-19 severity in naturally infected individuals

**Figure 1:**
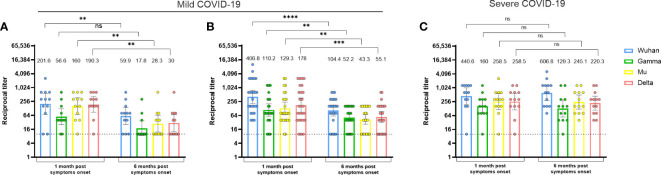
The durability of cross-reactive anti-RBD antibody responses upon natural infection is impacted by disease severity. Anti-RBD antibody titers against the Wuhan, Gamma, Mu, and Delta variants, in naturally-infected individuals who developed mild **(A, B)** or severe disease **(C)**. Each circle represents an individual sample. The height of the bars and the numbers over them indicate the geometric mean titer. The 95% CI is also shown. The Wilcoxon test was performed. The dotted line represents the limit of detection of the assay. In **(A)**, the Javeriana cohort is shown (n=12). In **(B)**, the Andes cohort is shown (1-month n=26 and 6-month PSO n=21). In **(C)**, the El Rosario cohort is shown (n=13). ns, Not statistically significant. *p-value <0.05; **p-value <0.01; ***p-value <0.001.

We first evaluated the durability of RBD-specific antibodies against the Wuhan, Gamma, Mu, and Delta variants in naturally infected individuals, according to disease severity. One-month PSO in individuals with mild (**Figures 1A, B**) or severe disease (**Figure 1C**), the antibody response against the Wuhan variant was predominant. However, most of the participants also showed RBD recognition for the viral variants, indicative of a cross-reactive antibody response. Interestingly, while RBD-specific antibody titers decreased (3.3 to 6.3-fold) at 6 months PSO in donors who developed mild disease (**Figures 1A, B**), the individuals who suffered severe disease maintained this response over time (**Figure 1C**), and indeed, antibody titers for the Wuhan variant tended to increase at 6 months PSO in them (**Figure 1C**). Our results showed that mild COVID-19 subjects reduced the RBD-specific antibody levels at 6 months PSO compared to 1-month PSO, but subjects who presented severe COVID-19 maintained antibody levels, suggesting that the durability of anti-RBD antibodies upon natural infection depends on disease severity.

### 3.3 Divergent humoral response against the Mu and Gamma variants

**Figure 2:**
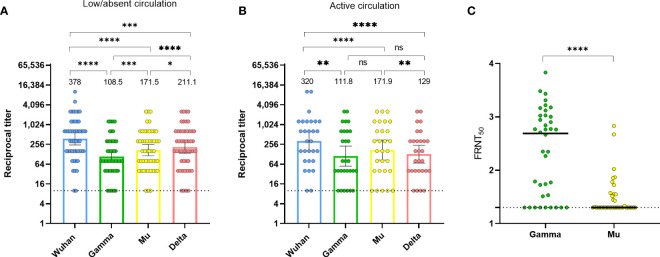
Divergence between the binding and neutralization potential of human sera against SARS-CoV-2 Mu and Gamma. **(A)** Anti-RBD antibody titers against the Wuhan ancestral strain, as well as Gamma, Mu, and Delta variants, in naturally-infected individuals (n=50, both mild and severe disease) at 1-month PSO between June and December 2020 (period of low/absent circulation of Gamma and Mu). **(B)** Anti-RBD antibody titers against the Wuhan ancestral strain, as well as Gamma, Mu, and Delta variants in samples from 29 individuals (Los Andes cohort) who were seronegative up to December 2020 and seroconverted 6 months later in May 2021, during an active circulation of Gamma and Mu variants. Each circle represents an individual sample. The height of the bars and the numbers over them indicate the geometric mean titer. The 95% CI is also shown. The Friedman test was performed. ns, Not statistically significant. **(C)** Neutralization of infectious SARS-CoV-2 by human sera was determined by Focus Neutralization Reduction Test (FRNT). FRNT_50_ values for SARS-CoV-2 Gamma and Mu were determined by non-linear regression analysis for SARS-CoV-2 naturally-infected subjects (samples at 1-month PSO). Each circle represents an individual sample. The bars indicate the geometric mean. The Wilcoxon test was performed. The dotted lines represent the limit of detection of the assays. *p-value <0.05; **p-value <0.01; ***p-value <0.001; ****p-value <0.0001.

In Colombia, in 2020, the circulation of the Gamma, Mu, and Delta variants was absent or low, and then, during 2021 these variants circulated, with Mu predominating during the third wave of the pandemic (21). Thus, we aimed to further evaluate the anti-RBD antibody cross-recognition in naturally-infected individuals during these two periods using the HAT. Despite the low or absent circulation of these viral variants (Gamma, Mu, and Delta) in the first period, there was antibody cross-recognition of all variants, with Delta exhibiting the highest recognition. Notably, Mu exhibited a significantly higher antibody cross-recognition than the Gamma variant (**Figure 2A**). During the period of active circulation of Gamma and Mu, the pattern seemed similar, although differences between Gamma and Mu did not differ statistically (**Figure 2B**). We were also able to evaluate the RBD antibody binding in serum from an individual with a confirmed infection by the Gamma variant, obtained 1-month PSO. Expectedly, we obtained high antibody titers against Gamma; although there was some level of cross-recognition for the Wuhan, Mu, and Delta variants, the antibody titers for these variants were lower than for Gamma (**Supplementary Figure 3**). These results further confirm the reproducibility and specificity of the HAT, as well as the cross-reactive nature of anti-RBD antibodies following natural infection with SARS-CoV-2. Moreover, these results suggest that natural infection with heterologous viruses elicits binding antibodies that are more cross-reactive for the RBD of the Mu than for the RBD of the Gamma variant.

Importantly, while we observed a higher antibody binding to Mu than to Gamma (**Figures 2A, B**), previous studies have shown that the Mu variant has higher antibody evasion than Gamma when evaluated by neutralizing assays (12). Thus, to extend the findings obtained by the HAT, we evaluated the neutralizing capacity of antibodies against the Gamma and Mu variants in serum samples obtained at 1-month PSO from SARS-CoV-2 naturally-infected individuals (**Figure 2C**). Interestingly, despite the higher antibody binding for the Mu variant (**Figures 2A, B**), the antibodies against Mu had a lower neutralizing capacity relative to Gamma (**Figure 2C**). Moreover, we observed a weak positive correlation between the neutralizing and binding antibodies for both variants (rho=0.30, p-value=0.02 for Gamma; and rho=0.30, p-value=0.03 for Mu). Thus, there is a weak correlation and a different pattern of response between the binding and neutralizing antibodies against the Mu and Gamma variants in Colombian individuals with natural SARS-CoV-2 infection.

### 3.4 Vaccination induces a SARS-CoV-2 specific antibody response, and its durability depends on previous virus exposure

We also evaluated antibody levels before and after vaccination. A first analysis including 57 individuals from cohort 2 recruited by the Javeriana, indicated that vaccination induced an increase in antibody titers at 15 – 30 days after receiving the second dose of the BNT162b2 vaccine, but they decreased at 6 months post-vaccination, showing at this time point similar titers to the pre-vaccination period (**Supplementary Figure 4A**). On the other hand, 38 individuals from cohort 2 recruited by El Rosario who received a third dose of the vaccine exhibited high antibody titers 1-3 months after the boost, which were comparable to those observed 15-30 days after the second vaccine dose (**Supplementary Figure 4B**). These results are consistent with a transitory boosting effect on the humoral response by sequential immunizations against SARS-CoV-2.

**Figure 3:**
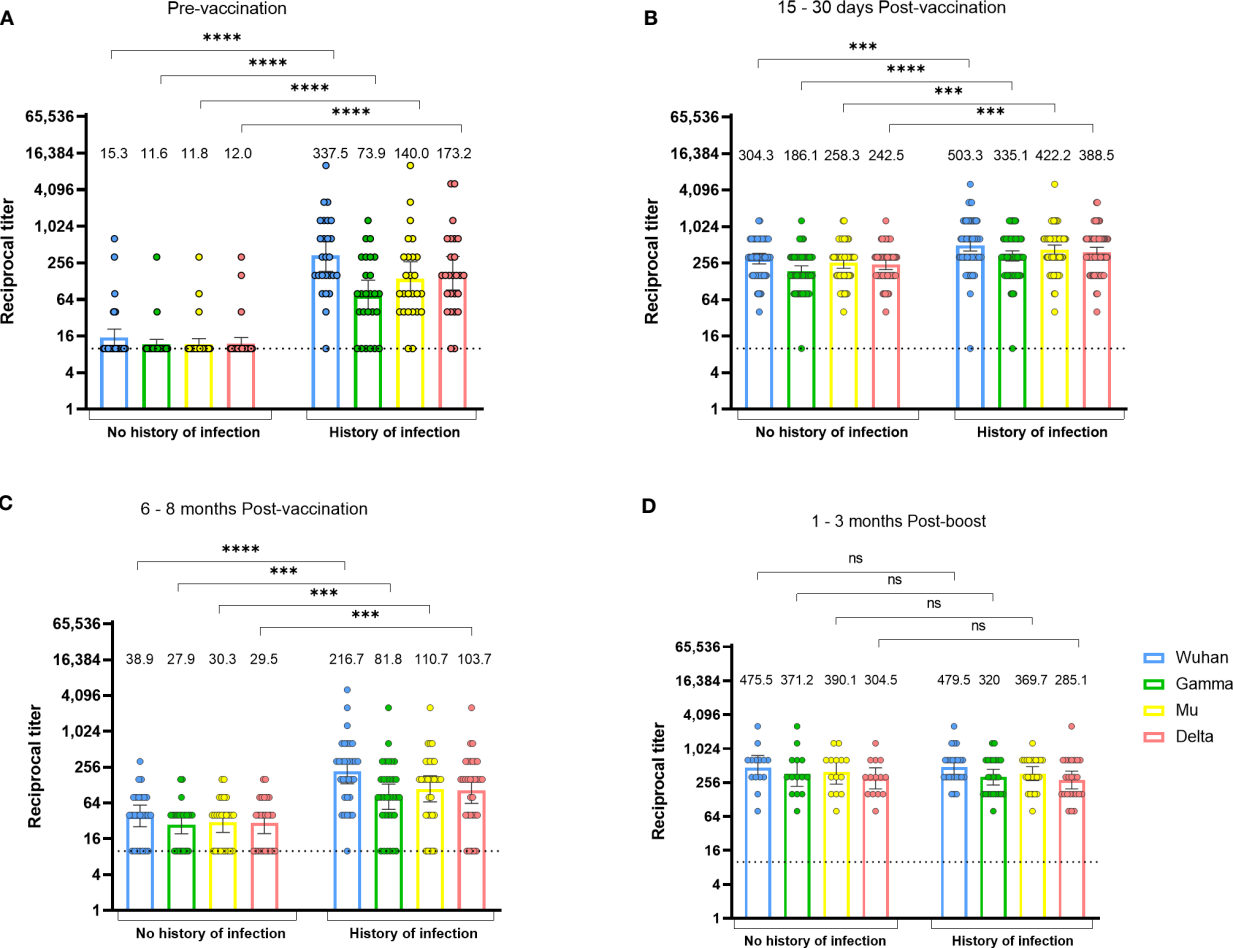
The durability of vaccine-induced anti-RBD antibodies depends on the history of natural infection. Anti-RBD antibody titers against the Wuhan, Gamma, Mu, and Delta variants, in BNT162b2-vaccinated individuals with or without history of natural infection. Data from individuals from the Javeriana cohort 2 (n=57) at the pre-vaccination **(A)**, 15-30 days post-vaccination **(B)**, and 6-8 months post-vaccination period **(C)**, or 1-3 months after a vaccine boost in individuals from the Rosario cohort 2 (n=38; **D**), are shown. Each circle represents an individual sample. The height of the bars and the numbers over them indicate the geometric mean titer. The 95% CI is also shown. The Mann-Whitney test was performed. The dotted line represents the limit of detection of the assay. ns, Not statistically significant. ***p-value <0.001; ****p-value <0.0001.

Next, we evaluated the antibody response pattern according to the antecedent of SARS-CoV-2 natural infection before receiving the first vaccine dose (**Figure 3**). As expected, individuals with a preceding infection had detectable antibody titers that cross-recognized all SARS-CoV-2 variants before vaccination, while most individuals without a preceding infection had undetectable anti-RBD antibodies (**Figure 3A**). Of note, in a few individuals that did not have a history of infection before vaccination and had a negative IgG nucleoprotein test, we could detect low levels of HAT antibodies against the viral variants RBD (**Figure 3A**), which may be indicative of a previous infection that was not detected by the nucleoprotein IgG assay, or of potential cross-reactivity with other HCoVs.

One month after the second vaccine dose, individuals with a history of infection generated higher levels of anti-RBD antibodies relative to those without a history of infection (**Figure 3B**). At 6 months after the second dose, antibody titers in individuals without a history of infection returned to almost undetectable levels, but those with previous infection were still able to maintain antibody titers comparable to those observed 1 month after the second dose (**Figure 3C**). There were no differences in antibody titers after a third vaccine dose when comparing those with and without pre-vaccination infection (**Figure 3D**). These results indicate that the durability of the anti-RBD response to vaccination depends on the history of pre-vaccination SARS-CoV-2 infection and a vaccine boost. Finally, as seen after natural infection, a higher cross-recognition of the Mu versus the Gamma variant was observed after vaccination (**Figures 3B–D**).

### 3.5 SARS-CoV-2 BTIs are associated with lower levels of vaccine-induced antibodies against viral variants

**Figure 4:**
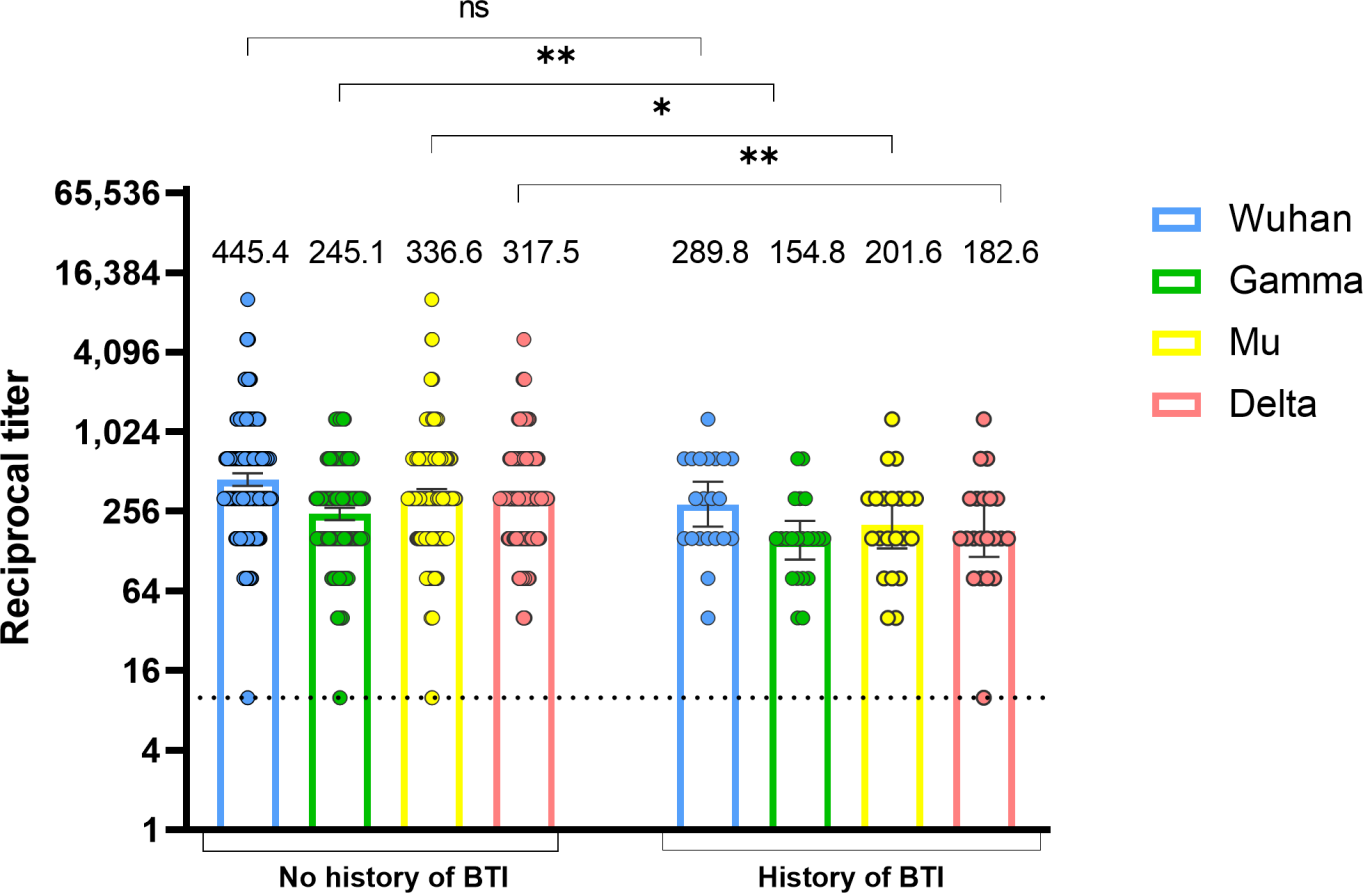
Lower vaccine-induced anti-RBD antibody titers are associated with the development of SARS-CoV-2 BTI. Anti-RBD antibody titers against the Wuhan, Gamma, Mu, and Delta variants were measured at 15-30 days after the second BNT162b2 vaccine dose in individuals with or without a history of BTI (n=21). BTI were determined by clinical records or seroconversion in nucleoprotein antibodies measured 6-8 months after the second vaccine dose. Each circle represents an individual sample. The height of the bars and the numbers over them indicate the geometric mean titer. The 95% CI is also shown. The Mann-Whitney test was performed. The dotted line represents the limit of detection of the assay. ns, Not statistically significant; BTI, Breakthrough infection. *p-value <0.05; **p-value <0.01.

To determine whether vaccine-elicited antibody levels measured 15-30 days after the second vaccine dose are associated with protection against SARS-CoV-2 infection, we classified individuals according to the occurrence or not of a BTI up to 6-8 months after vaccination. BTI that occurred after vaccination were identified based on clinical records of mild infections and seroconversion in SARS-CoV-2 nucleoprotein-specific IgG between samples taken 15-30 days and 6-8 months after the second vaccine dose. As shown in **Figure 4**, individuals with BTIs had lower antibody titers against the Gamma, Mu, and Delta variants in comparison with individuals without BTI. No statistical differences were observed for antibodies against the Wuhan variant (**Figure 4**). These results indicate that the levels of RBD-specific antibodies post-immunization are associated with protection against SARS-CoV-2 BTI.

### 3.6 The BNT162b2 vaccine induces virus-specific CD4^+^ T cells that cross-react with viral variants

**Figure 5:**
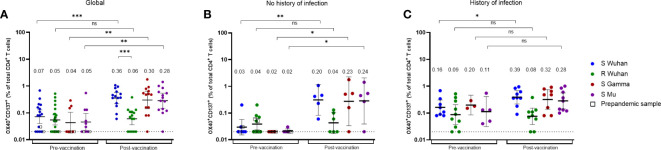
The magnitude of vaccine-induced SARS-CoV-2 specific CD4^+^ T cell responses depends on the history of natural infection. Frequencies of Wuhan S or R-specific CD4^+^ T cells, as well as Gamma and Mu S-specific CD4^+^ T cells, evaluated by the AIM assay (OX40^+^CD137^+^ CD4^+^ T cells), at the pre-vaccination or post-vaccination period, in all the individuals analyzed **(A)**, or in those without **(B)** or with history of natural infection **(C)**. Each symbol represents an individual sample and open squares represent samples obtained from individuals before the beginning of the pandemic. Cells were stimulated with one or more peptide pools depending on the availability of cells per sample. The geometric mean and 95% CI are shown. The Mann-Whitney test was performed. The dotted line represents the threshold of positivity. ns, Not statistically significant. *p-value <0.05; **p-value <0.01; ***p-value <0.001.

We next evaluated the CD4^+^ T cell response in individuals before and after receiving the BNT162b2 vaccine. Using the AIM assay, we measured the frequency of SARS-CoV-2-specific CD4^+^ T cells (defined as OX40^+^ CD137^+^ cells) for S peptides of the Wuhan, Gamma, and Mu variants, in 20 donors pre-vaccination, and 16 of them 1 - 2 months after the second vaccine dose (**Figure 5**). In the pre-vaccination group, we included four samples of individuals (represented with uncolored squares) obtained before the onset of the pandemic (**Figure 5**). Of note, in the cohort of individuals in whom we performed cellular analyses, 13 out of 20 donors exhibited an antibody response against the Wuhan, Gamma, or Mu variants, indicative of previous coronavirus exposure.

We first performed an analysis with all the donors included. As expected, the frequencies of SARS-CoV-2-specific CD4^+^ T cells were low during the pre-vaccination period, while the frequencies of S-specific, but not R-specific CD4^+^ T cells increased upon vaccination (**Figure 5A**). Importantly, the CD4^+^ T cell response cross-recognized peptides from the Gamma and Mu variants (**Figure 5A**). We next assessed the impact of natural infection before vaccination on the dynamics of the SARS-CoV-2-specific CD4^+^ T cell response. We classified the individuals as those with or without a history of natural infection, based on the detection of nucleoprotein-specific IgG antibodies in samples before and after vaccination. Of note, we observed detectable SARS-CoV-2-specific CD4^+^ T cells for the Wuhan S and R proteins in the pre-vaccination samples of some individuals without a history of natural infection and in some pre-pandemic samples (**Figure 5B**). This result might be explained by undetected asymptomatic SARS-CoV-2 infections and/or cross-reactive responses to previous infections with HCoVs. Interestingly, while unexposed individuals exhibited an increase in the frequencies of Wuhan, Gamma, and Mu S-specific CD4^+^ T cells upon vaccination (**Figure 5B**), this was not the case for individuals with a history of natural infection, since they exhibited high frequencies of Wuhan, Gamma, and Mu S-specific CD4^+^ T cells during the pre-vaccination period, and only had a modest increase in the frequency of Wuhan S-specific CD4^+^ T cells upon vaccination (**Figure 5C**). These results indicate that the dynamic of the SARS-CoV-2-specific CD4^+^ T cell response is dependent on the history of SARS-CoV-2 exposure.

### 3.7 Dynamics of the memory profile of SARS-CoV-2-specific CD4^+^ T cells according to the history of viral exposure

To assess the quality of the SARS-CoV-2-specific CD4^+^ T cell response, we analyzed the memory phenotype (CD45RA and CCR7) of AIM^+^ (OX40^+^ CD137^+^) CD4^+^ T cells in response to peptide stimulation. The analysis of all the individuals included showed that peptide-responding cells at the pre-vaccination assessment had predominantly a central memory phenotype (T_CM_; CD45RA^-^ CCR7^+^), followed by an effector memory phenotype (T_EM_; CD45RA^-^ CCR7^-^), and these profiles were higher than those found in bulk CD4^+^ T cells (**Supplementary Figure 5A**). This pattern was similar for cells specific for S-derived peptides from Wuhan, Gamma, and Mu variants, as well as for Wuhan R-derived peptides (**Supplementary Figure 5A**). Upon vaccination, SARS-CoV-2-specific CD4^+^ T cells were enriched in a T_EM_ and T_CM_ phenotype relative to bulk CD4^+^ T cells, but the T_CM_ profile (suggestive of long-lived responses) predominated along CD4^+^ T cells specific for the Wuhan, Gamma, and Mu variants (**Supplementary Figure 5B**). Low frequencies of naïve/T stem cell memory (CD45RA^+^ CCR7^+^) and T_EMRA_ (CD45RA^+^ CCR7^-^) SARS-CoV-2-specific CD4^+^ T cells were observed at both time points (**Supplementary Figure 5**).

**Figure 6:**
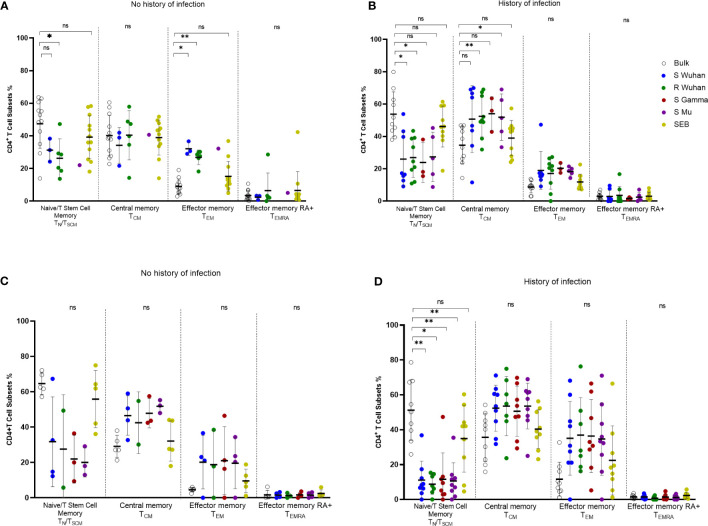
Changes in the phenotype of SARS-CoV-2 specific CD4^+^ T cells according to the history of antigen exposure. Phenotype of SARS-CoV-2-specific CD4^+^ T cells evaluated by the AIM assay (OX40^+^CD137^+^ CD4^+^ T cells) in pre-vaccination **(A, B)** or post-vaccination **(C, D)** samples from individuals without **(A, C)** or with history of SARS-CoV-2 natural infection **(B, D)**. Each symbol represents an individual sample. The geometric mean and 95% CI are shown. The Kruskal-Wallis and Dunn *post-hoc* tests were performed. ns, Not statistically significant. *p-value <0.05; **p-value <0.01.

We observed that during the pre-vaccination period, some individuals without a history of natural infection had detectable SARS-CoV-2-specific CD4^+^ T cells (**Figure 5B**). Thus, we aimed to evaluate the phenotype of these potentially SARS-CoV-2 cross-reactive CD4^+^ T cells. Interestingly, SARS-CoV-2-specific CD4^+^ T cells, including those specific for R-derived peptides, in individuals without a history of natural infection were enriched in a T_EM_ phenotype, when compared with bulk CD4^+^ T cells, while a high proportion also exhibited a T_CM_ phenotype (**Figure 6A**). These data support that these cross-reactive cells are part of the memory compartment. On the other hand, SARS-CoV-2-specific CD4^+^ T cells at the pre-vaccination time point from donors with a history of natural infection were enriched in a T_CM_ phenotype, further supporting the notion that they are long-lived memory cells (**Figure 6B**). Finally, we evaluated the changes in the memory phenotype of SARS-CoV-2-specific CD4^+^ T cells upon vaccination. Interestingly, we noted that in individuals without a history of SARS-CoV-2 infection, the phenotype of SARS-CoV-2-specific CD4^+^ T cells tended to augment in a T_CM_ profile after vaccination, relative to pre-vaccination values (**Figures 6A, C**). On the other hand, in individuals with a history of infection, the proportion of T_EM_ among SARS-CoV-2-specific CD4^+^ T cells tended to increase upon vaccination (**Figures 6B, D** **)**, and this increment was statistically significant for S Wuhan-specific cells (p-value=0.03). These data indicate that the number of exposures to the viral antigens induces changes in the memory profile of SARS-CoV-2-specific CD4^+^ T cells.

## 4 Discussion

In this study, we evaluated the SARS-CoV-2-specific humoral and the CD4^+^ T cell response to selected viral variants upon natural infection and following vaccination in a Colombian population. Our data show that the immune response induced either by natural infection with the Wuhan variant and/or vaccination generates SARS-CoV-2-specific antibodies and CD4^+^ T cells that cross-react with the VOIs/VOCs Gamma, Mu, and Delta. In addition, we evidenced that the durability of anti-RBD antibodies is associated with the severity of COVID-19 in individuals with a history of natural infection, while the humoral response induced by vaccination changes according to the number of exposures to viral antigens. Lower levels of antibody titers against viral variants after vaccination were observed in individuals who developed a BTI. Also, the frequency of S-specific CD4^+^ T cell response is influenced by previous natural infection. Interestingly, we identified individuals with no history of SARS-CoV-2 infection in whom their CD4^+^ T cells recognized predominantly R-derived peptides which exhibited a central and effector memory phenotype, suggesting cross-reaction with endemic HCoVs. Finally, S-specific CD4^+^ T cells found upon vaccination also showed a central memory phenotype, suggestive of long-lived responses.

A primary objective of our study was to evaluate the durability of the humoral response upon natural infection and/or vaccination, and the influence of sequential antigen exposures. Consistent with previous studies, our results showed that the durability of the SARS-CoV-2 RBD-specific antibodies in naturally infected individuals depends on the severity of the disease (22, 23). We observed that individuals who had a mild SARS-CoV-2 infection had high titers of RBD-specific antibodies at 1-month PSO, but they decreased at 6 months PSO. However, it has been reported that despite the decrease in antibody titers, they can be detected in most individuals up to 15 months PSO, including neutralizing antibodies (24–26). In contrast, convalescent donors who experienced severe disease maintained high antibody titers, probably related to an increased or more sustained antigen exposure during acute infection in comparison with individuals who experienced mild disease (27). In addition, previous studies have shown that individuals with severe disease develop higher titers of low-affinity antibodies in comparison with individuals with mild disease (28, 29). This lower affinity may impair the neutralization capacity of these antibodies, which in turn could contribute to more severe disease. Another possibility is that individuals who suffered a severe SARS-CoV-2 infection are poised to generate robust extrafollicular B cell responses, that have been shown to correlate with anti-SARS-CoV-2 antibody titers (30). In the case of vaccine-induced antibodies, we observed that mRNA-based vaccines induced an increase in antibody titers 15 – 30 days after receiving the second dose, but these levels decreased at 6 months, as occurred in individuals who experienced a mild SARS-CoV-2 infection. However, a third dose of the vaccine caused a rise in antibody titers, indicative of a boosting effect on the humoral response upon sequential antigen exposure (17, 31). Notably, the dynamics of the antibody response upon vaccination depended on the previous SARS-CoV-2 exposure. As such, individuals with hybrid immunity were able to maintain high anti-RBD antibody titers up to 6 months post-vaccination. These data are in line with previous studies showing that hybrid immunity potentiates the magnitude, breadth, and neutralizing activity of SARS-CoV-2-specific antibodies (17, 32, 33). Thus, hybrid immunity seems to better promote the maintenance of the SARS-CoV-2-specific antibody response than vaccination alone. Nonetheless, unexposed individuals benefit from sequential vaccine boosters.

The emergence of viral variants has been one of the major concerns affecting the measures implemented to contain the SARS-CoV-2 pandemic. Hence, it is important to determine the capacity of antibodies to cross-recognize other viral variants distinct from those responsible for an infection episode or used in a particular vaccine. Here we evaluated responses to the Gamma, Mu, and Delta variants as they contributed to a significant disease burden during their active circulation in Colombia. In agreement with previous studies (16, 34, 35), the Wuhan variant showed the highest antibody titers in all our study cohorts, followed by the Delta variant. Importantly, our results also indicated that the Mu variant is recognized at higher levels than the Gamma variant both after natural infection and vaccination. This seems at odds with what has been shown for neutralizing antibodies (11, 12, 14, 15, 36): In studies in which they have been directly compared, the levels of neutralizing antibodies induced by natural infection (11–14), as well as vaccination (15, 36–40), are higher against Gamma than against Mu, suggesting that the latter has a particular immune evasion capacity. Moreover, serum from hamsters previously infected with an early isolate of SARS-CoV-2 neutralized Mu to a low degree (39). Notwithstanding, hamsters were partially protected from rechallenge with the Mu variant, suggesting that the immune response to the first infection was sufficient to protect against Mu (39). On the other hand, although vaccination is efficient against infection with the Mu variant (41), BTIs with Mu have been reported (42). Here we further demonstrate that, although naturally-induced binding antibodies against Mu were higher than for Gamma, these antibodies possess a poor neutralizing capacity, consistent with previous reports (11–14). However, we observed that binding antibodies were lower in vaccinated individuals who developed BTI than in those who did not, suggesting that these antibodies can protect against COVID-19 despite a relatively poor neutralizing capacity. Recent studies have shown that relatively high protection against symptomatic COVID-19 can be achieved at low serum neutralizing antibody titers in vaccination settings (43). A good correlation has been found previously between HAT and neutralizing antibody titers to the Alpha, Beta, Delta, and Wuhan variants (19, 44). The Gamma and Mu RBD (that both share the E484K, N501Y mutations) may represent a particular example for which a poor correlation exists between binding and neutralizing antibodies. Collectively, these observations highlight the complexity and dynamic role of humoral immunity against SARS-CoV-2 emerging variants. The effector mechanisms of protective SARS-CoV-2-specific antibodies remain to be further defined.

At the level of the cellular immune response, we assessed the frequency of SARS-CoV-2-specific CD4^+^ T cells against the Wuhan, Gamma, and Mu variants, before and after BNT162b2 vaccination. To our knowledge, this is the first study that evaluates the CD4^+^ T cell response against the Mu variant after natural infection (pre-vaccination samples) in a period of active circulation of this variant, and the second after vaccination (4). The S-specific CD4^+^ T cell response induced by vaccination cross-recognized peptides from the Wuhan, Gamma, and Mu variants, in line with previous studies (3, 4, 16). This cross-recognition phenomenon of the SARS-CoV-2 variants is likely a result of the large conservation of T cell epitopes (3, 4). In the case of CD4^+^ T cells, it has been reported that 93% of the epitopes are fully conserved in the SARS-CoV-2 variants (3). Interestingly, R-derived peptides from the Wuhan variant showed AIM^+^ CD4^+^ T cells during the pre-vaccination period, suggesting either asymptomatic SARS-CoV-2 infections or past infections with HCoVs (6, 45). These findings led us to evaluate if preexisting immunity could modulate the CD4^+^ T cell response induced by vaccination. In individuals without previous natural infection, the CD4^+^ T cell response to the S proteins exhibited an increase in magnitude upon vaccination. However, except for the Wuhan S peptides, this was not the case for individuals with a history of natural infection, since they already exhibited high frequencies of cells reactive to Gamma and Mu variants, that were not boosted by the vaccine. In this regard, it has been reported that although SARS-CoV-2 exposure does not impact the magnitude of S-specific CD4^+^ T cells upon COVID-19 vaccination, it can induce functionally distinct antigen-specific cells relative to naïve individuals, and this factor might contribute to disease protection (46). Moreover, SARS-CoV-2-specific CD8^+^ T cells are also modulated by hybrid immunity (47, 48), but the clinical significance of this aspect remains to be addressed.

We also determined the memory phenotype of S-specific CD4^+^ T cells according to the infection background. Like a previous report (45), cross-reactive SARS-CoV-2-specific CD4^+^ T cells from donors without a history of infection exhibited a T_CM_ and T_EM_ phenotype, indicating that they are most probably memory cells elicited by previous exposure to HCoVs. In addition, the SARS-CoV-2-specific CD4^+^ T cell response from individuals with a history of natural infection, displayed primarily a T_CM_ phenotype, like previous reports (49, 50). However, it was interesting to observe that antigen re-exposure with vaccination in this group of individuals induced an increase in T_EM_ responses, comparable to what has been observed for CD8^+^ T cells (48). While the development of T_CM_ responses could contribute to longer-lived CD4^+^ T cell responses, as well as lymph node homing that might contribute to the promotion of the B cell antibody response (49), terminal differentiation upon iterative antigen exposures might lead to a decrease in the lifespan of SARS-CoV-2-specific memory CD4^+^ T cells. These data suggest that sequential immunizations impact the phenotypic (and likely the functional) profile of SARS-CoV-2-specific T cells. Thus, our results have implications for the design of vaccine schemes and boosters.

This study has limitations. The HAT assay measures total antibodies, and we did not determine the isotypes of the antibodies in the samples. Additionally, we were not able to evaluate the kinetics of antibodies at 6 months after the second vaccine dose in individuals from El Rosario cohort 2. On the other hand, the sample size for the analysis of the CD4^+^ T cell response was low, and for these individuals, we were not able to identify BTIs or evaluate samples at late time points after vaccination. Moreover, the number of cells recovered from some volunteers was low, limiting the evaluation of T cells specific for all peptide mega pools. Finally, the functionality of SARS-CoV-2-specific CD4^+^ T cells remains to be evaluated.

## 5 Conclusions

The humoral and CD4^+^ T cell response induced by natural infection and/or vaccination with the Wuhan variant can cross-recognize several viral variants of interest and concern that circulated in Colombia during the second and third waves that occurred in the country. The durability of RBD-specific antibodies induced by infection depends on the severity of the disease, while the humoral response induced by vaccination is modulated by the number of vaccine doses. The Gamma and Mu RBDs may be an example of a poor correlation between binding and neutralizing antibodies. Similarly, the frequency of vaccine-induced S-specific CD4^+^ T cells is influenced by previous natural infection. These results have important implications for vaccine design to prevent the dissemination of new viral variants in Colombia.

## Data availability statement

The original contributions presented in the study are included in the article/**Supplementary Material**. Further inquiries can be directed to the corresponding author.

## Ethics statement

The studies involving human participants were reviewed and approved by Ethics committee of the School of Medicine, Pontificia Universidad Javeriana, Universidad de Los Andes, and Universidad del Rosario. All the procedures followed the principles of the Declaration of Helsinki. The patients/participants provided their written informed consent to participate in this study.

## Author contributions

MF, JA, JC-R, JG, SV-B, BA, MC, and CC-R conceptualized and designed the studies. FM performed the HAT and flow cytometry experiments for the phenotypic characterization of T cell subsets, analyzed the data, constructed the figures, and contributed to writing the first draft of the manuscript. JB, AD, and AnG designed and performed the neutralizing antibody experiments. JC-R, BA, JG, CR-S, YA-A, LM-S, NM, CC-R, SV-B, and MC contributed to the recruitment of participants. YA-A, JG, CR-S, CG-R, and JC-R contributed to obtaining funding. JM, AlG, AS, and DW contributed with experimental design and key reagents of T cell experiments. FP-C supervised the T cell experiments and contributed to writing the first version of the paper. JA and MF wrote and obtained funding for the project and directed the work and writing of the paper. All authors reviewed and edited the manuscript. All authors contributed to the article and approved the submitted version.
