# Corrigendum: Cross-reactive humoral and CD4^+^ T cell responses to Mu and Gamma SARS-CoV-2 variants in a Colombian population

**DOI:** 10.3389/fimmu.2023.1274753

**Published:** 2023-08-16

**Authors:** Fabiola Martel, Juliana Cuervo-Rojas, Juana Ángel, Beatriz Ariza, John Mario González, Carolina Ramírez-Santana, Yeny Acosta-Ampudia, Luisa Murcia-Soriano, Norma Montoya, Claudia Cecilia Cardozo-Romero, Sandra Liliana Valderrama-Beltrán, Magda Cepeda, Julio César Castellanos, Carlos Gómez-Restrepo, Federico Perdomo-Celis, Andreu Gazquez, Alexandria Dickson, James D. Brien, José Mateus, Alba Grifoni, Alessandro Sette, Daniela Weiskopf, Manuel A. Franco

**Affiliations:** ^1^ Institute of Human Genetics, School of Medicine, Pontificia Universidad Javeriana, Bogotá, Colombia; ^2^ Department of Clinical Epidemiology and Biostatistics, School of Medicine, Pontificia Universidad Javeriana, Bogotá, Colombia; ^3^ Clinical Laboratory Science Research Group, Clinical Laboratory, Hospital Universitario San Ignacio, Bogotá, Colombia; ^4^ Group of Basic Medical Sciences, School of Medicine, Universidad de Los Andes, Bogotá, Colombia; ^5^ Center for Autoimmune Diseases Research (CREA), School of Medicine and Health Sciences, Universidad del Rosario, Bogotá, Colombia; ^6^ Hospital Universitario Mayor-Méderi, Universidad El Rosario, Bogotá, Colombia; ^7^ Head Clinical Laboratory Unit, Clínica del Occidente, Bogotá, Colombia; ^8^ Division of Infectious Diseases, Department of Internal Medicine. School of Medicine, Pontificia Universidad Javeriana, Hospital Universitario San Ignacio Infectious Diseases Research Group, Bogotá, Colombia; ^9^ General Direction, Hospital Universitario San Ignacio, Bogotá, Colombia; ^10^ Department of Molecular Microbiology and Immunology, Saint Louis University School of Medicine, St. Louis, MO, United States; ^11^ Center for Infectious Disease and Vaccine Research, La Jolla Institute for Immunology, La Jolla, CA, United States; ^12^ Department of Medicine, Division of Infectious Diseases and Global Public Health, University of California, San Diego, La Jolla, CA, United States

**Keywords:** SARS-CoV-2, variants, natural infection, vaccination, antibody, CD4+ T cell, hybrid immunity, breakthrough infections

In the published article, there was an error in affiliation 5. Instead of “Center for the Study of Autoimmune Diseases Research (CREA), School of Medicine and Health Sciences, Universidad del Rosario, Bogota´, Colombia”, it should be “Center for Autoimmune Diseases Research (CREA), School of Medicine and Health Sciences, Universidad del Rosario, Bogotá, Colombia”.

The authors apologize for this error and state that this does not change the scientific conclusions of the article in any way. The original article has been updated.

