# Supplementary material for: Cross-reactive humoral and CD4+ T cell responses to Mu and Gamma SARS-CoV-2 variants in a Colombian population
Source: Front Immunol. 2023 Jul 27;14:1241038. doi: 10.3389/fimmu.2023.1241038 (PMC10413264; doi:10.3389/fimmu.2023.1241038)
Supplement: Supplementary file 1 [file DataSheet_1.docx]

Supplementary Material

**Cross-reactive humoral and CD4^+^ T cell responses to Mu and Gamma SARS-CoV-2 variants in a Colombian population**

Fabiola Martel^1^, Juliana Cuervo-Rojas^2^, Juana Ángel^1^, Beatriz Ariza^3^, John Mario Gonzáles^4^, Carolina Ramírez-Santana^5^, Yenny Acosta-Ampudia^5^, Luisa Murcia-Soriano^6^, Norma Montoya^7^, Claudia Cardozo-Romero^3^, Sandra Valderrama-Beltrán^8^, Magda Cepeda^2^, Julio César Castellanos^9^, Carlos Gómez-Restrepo^2^, Federico Perdomo-Celis^1^, Andreu Gazquez^10^, Alexandria Dickson^10^, James D. Brien^10^, José Mateus^11^, Alba Grifoni^11^, Alessandro Sette^11,12^, Daniela Weiskopf^11^ and Manuel A. Franco^1^*

Corresponding author: Manuel Antonio Franco; [mafranco@javeriana.edu.co](mailto:mafranco@javeriana.edu.co)

**Resources table**

| **Antibodies** | **Fluorochrome** | **Clone** | **Manufacturer** | **Ref. No** | **Dose** |
| --- | --- | --- | --- | --- | --- |
| CD14 | V500 | M5E2 | BD | 561391 | 1:100 |
| CD19 | V500 | HIB19 | BD | 561121 | 1:100 |
| CD3 | Alexa Fluor 700 | UCHT1 | Biolegend | 300424 | 1:50 |
| CD4 | BV605 | RPA-T4 | BD | 562658 | 1:50 |
| CD8 | BV650 | RPA-T8 | Biolegend | 301042 | 1:50 |
| OX40/CD134 | PE/Cy7 | BER ACT35 | Biolegend | 350012 | 1:50 |
| CD137 | APC | 4-B4-1 | Biolegend | 309810 | 1:25 |
| CD45RA | BV421 | HI100 | Biolegend | 304130 | 1:50 |
| CD197 | FITC | G043H7 | Biolegend | 353216 | 1:50 |
| Live/Dead Viability | Aqua | - | Thermo Fisher | L34957 | 1:800 |

**Supplementary Table 1.** **Detailed information of antibodies used in the AIM assay of this study.**

**Supplementary Data**

**Supplementary Table 2. Description of PBMCs donor characteristics and demographics before and after mRNA-based BNT62b vaccine.**

| **Cohort Characteristics** | **Pre-vaccination** | **1 -2 months post-vaccination** |
| --- | --- | --- |
| **Number of donors** | 20 | 16 |
| **Age** | 33.5 (19 - 42) | 33.7 (19 - 42) |
| **Sex (M/F)** | (2/18) | (2/14) |
| **Sample collection date** | March 2021 | May - June 2021 |
| **# of positive Nucleoprotein antibody responders** | 8 | 5 |

**Supplementary Figures**


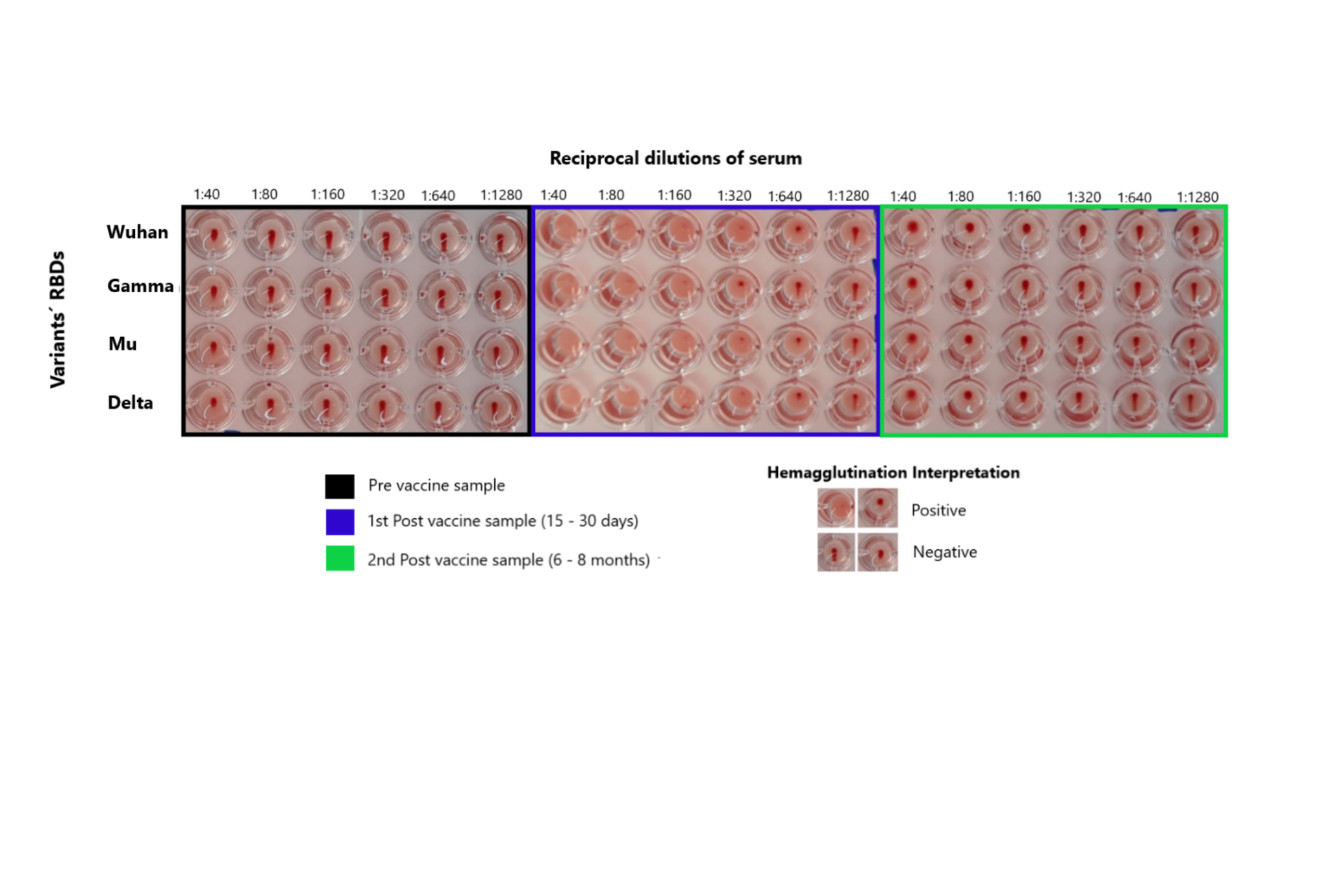


**Supplementary Figure 1. Hemagglutination test for the detection of antibodies against the receptor binding domain of SARS-CoV-2.** Example of an experiment measuring antibody titers in a pre-vaccine, and early and late post-vaccination samples against SARS-CoV-2 Wuhan, Gamma, Mu, and Delta variants. A positive HAT titer is defined as the last well in which O-negative red blood cells teardrop did not form. Partial teardrops are considered negative.


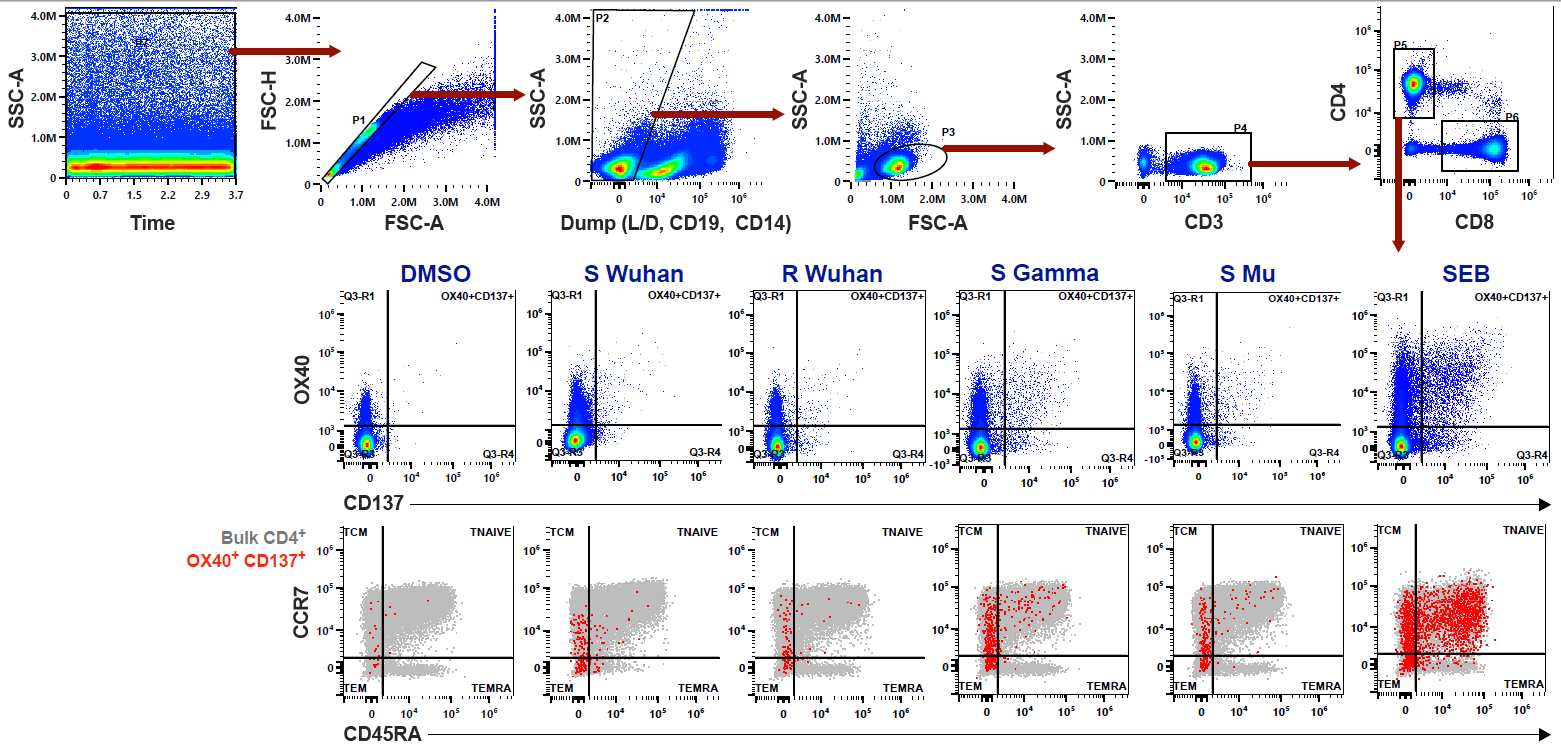


**Supplementary Figure 2. Gating strategy and representative CD4^+^ T cell response in the activation-induced marker (AIM) assay.** A post-vaccination sample from an individual with a history of natural infection is shown. PBMCs were gated by single cell determination. CD4^+^ T cells were defined as negative for the dump channel (Live/Dead Aqua, CD19, and CD14) and positive for CD3. Next, CD3^+^ CD4^+^ cells were gated according to AIM markers OX40 and CD137. Finally, the expression of the memory markers CCR7 and CD45RA was evaluated in AIM^+^ cells (OX40^+^ CD137^+^), to identify cells with a naïve/T stem cell memory (CCR7^+^ CD45RA^+^), central memory (CCR7^+^ CD45RA^-^), effector memory (CCR7^-^ CD45RA^-^), or TEMRA (CCR7^-^ CD45RA^+^) phenotype.


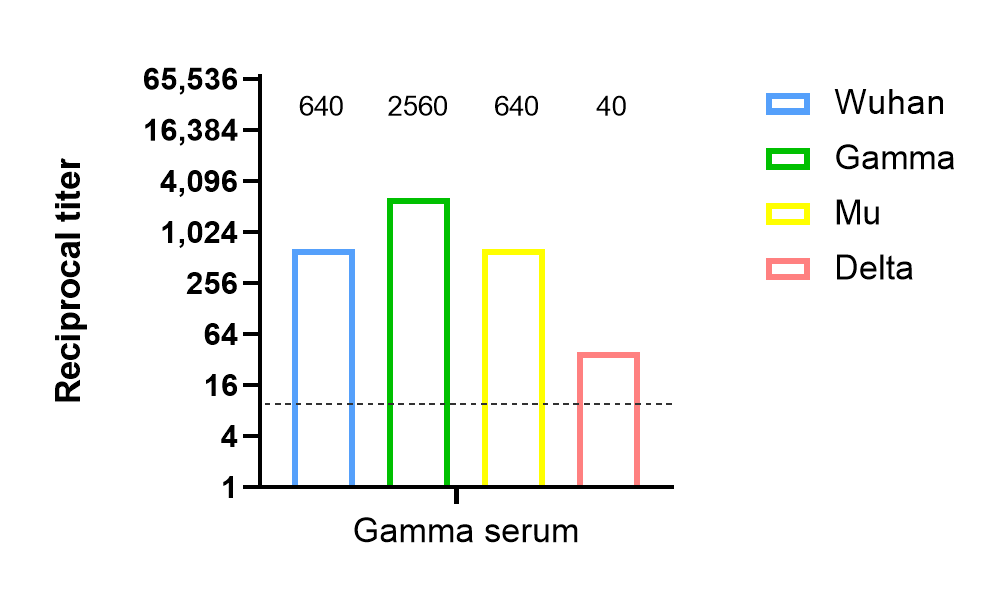


**Supplementary Figure 3. Strong antibody recognition of Gamma RBD in a naturally-infected individual.** Anti-RBD antibody titers against the Wuhan, Gamma, Mu, and Delta variants, in a naturally-infected individual with infection by the Gamma variant confirmed by PCR and sequencing. The height of the bars and numbers over them indicate the reciprocal titer for each variant. The dotted line represents the limit of detection of the assay.


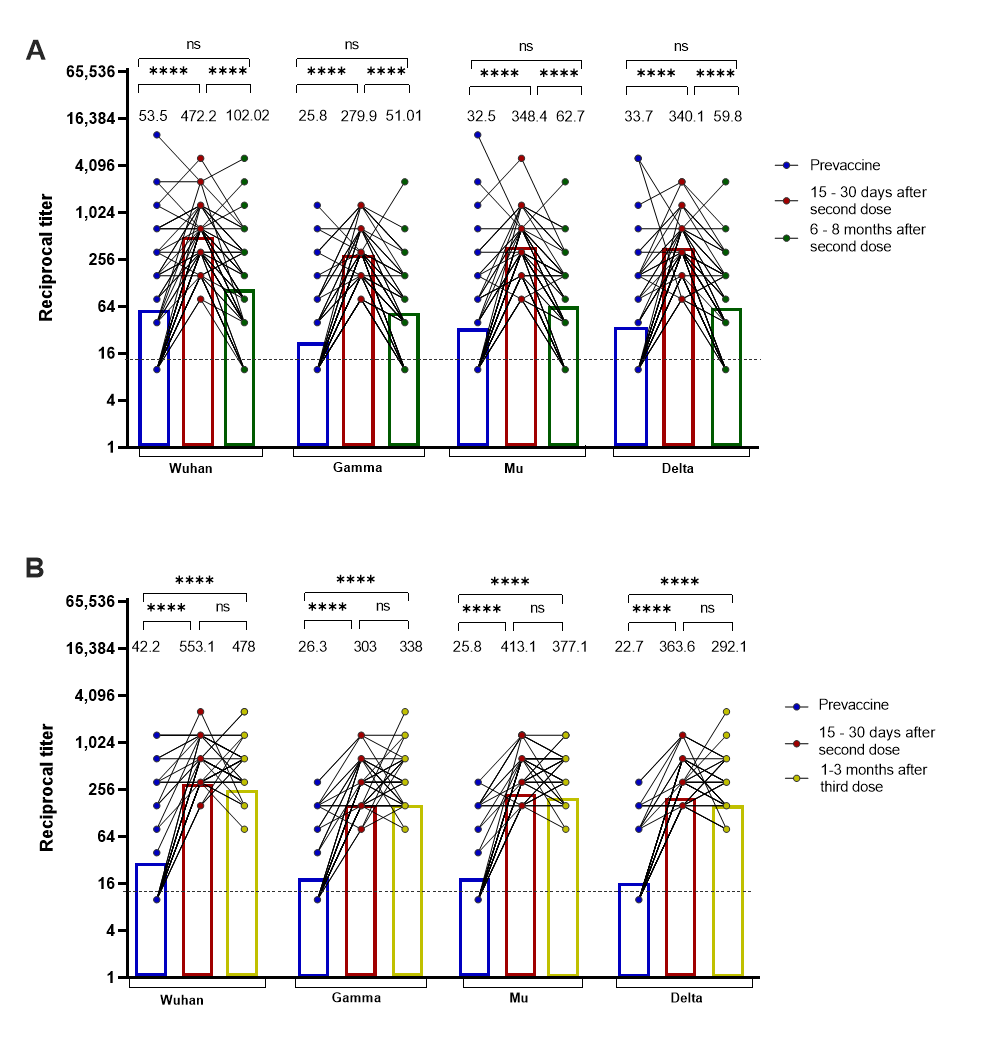


**Supplementary Figure 4. Dynamic anti-RBD antibody response upon vaccination.** Anti-RBD antibody titers against the Wuhan, Gamma, Mu, and Delta variants, in individuals who received two doses of the BNT162b2 vaccine in the Javeriana cohort (n=57; **A**), or who received a third dose in the El Rosario Cohort (**B**). Each circle and connecting line represent an individual paired sample. The height of the bars and numbers over them indicate the geometric mean titer. The 95% CI is also shown. The Friedman and Dunn post-hoc tests were performed. The dotted line represents the limit of detection of the assay. ns: Not statistically significant.


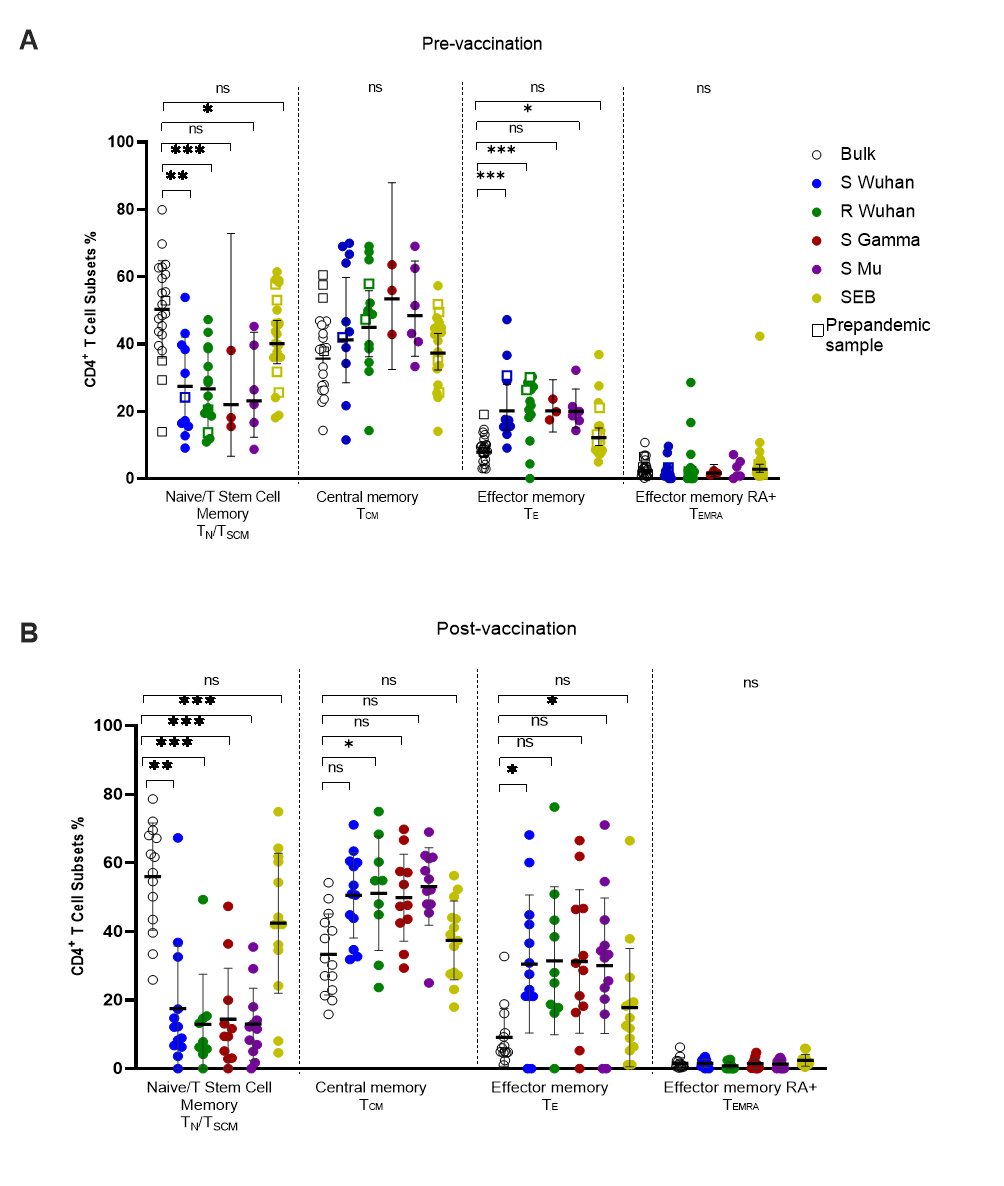


**Supplementary Figure 5. Memory phenotype of SARS-CoV-2-specific CD4^+^ T cells.** Phenotype of pre-vaccination (**A**) and post-vaccination (**B**) SARS-CoV-2-specific CD4^+^ T cells evaluated by the AIM assay (OX40^+^CD137^+^ CD4^+^ T cells). Each symbol represents an individual sample. The geometric mean and 95% CI are shown. The Kruskal-Wallis and Dunn post-hoc tests were performed. ns: Not statistically significant.
